# Exploring the Impact of Refractive and Ocular Residual Astigmatism on Stereopsis After Photorefractive Keratectomy in Patients With Myopic Astigmatism

**DOI:** 10.1155/joph/6140496

**Published:** 2026-01-07

**Authors:** Faezeh Fayaz, Mohsen Pourazizi, Payam Nabovati, Alireza Peyman, Pegah Noorshargh

**Affiliations:** ^1^ Department of Optometry, School of Rehabilitation Sciences, Rehabilitation Research Center, Iran University of Medical Sciences, Tehran, Iran, iums.ac.ir; ^2^ Department of Ophthalmology, Isfahan Eye Research Center, Isfahan University of Medical Sciences, Isfahan, Iran, mui.ac.ir; ^3^ Isfahan Eye Research Center, Isfahan University of Medical Sciences, Isfahan, Iran, mui.ac.ir

**Keywords:** astigmatism, depth perception, photorefractive keratectomy, stereopsis

## Abstract

**Background:**

To investigate the impact of refractive and ocular residual astigmatism (ORA) on stereopsis after photorefractive keratectomy (PRK) for the correction of myopic astigmatism.

**Methods:**

This prospective observational study was conducted on patients who underwent PRK for the treatment of myopia and myopic astigmatism using a Schwind Amaris 1050RS excimer laser system. Stereoacuity was measured using the Stereo Fly Test before and after the surgery. We evaluated the impact of pre‐op refractive and internal astigmatic components on stereopsis outcome after surgery. To calculate ORA, we calculated the vectorial difference between corneal plane refractive astigmatism and keratometric astigmatism.

**Results:**

Ninety‐six eyes from 96 patients including 70 females (72.9%) with a mean age of 29.83 ± 7.39 years were enrolled. The average stereoacuity measured in log units before and after PRK was 1.65 ± 0.09 and 1.63 ± 0.07, respectively (*p* = 0.17). Patients who showed stereopsis improvement had a higher mean cylindrical error compared to those who did not (*p* = 0.04). The average pretreatment ORA was 0.84 ± 0.37 in patients who experienced deterioration in stereopsis, 0.77 ± 0.35 in patients who maintained the same level, and 0.80 ± 0.35 in patients who showed improvement (*p* = 0.77).

**Conclusion:**

This study showed the potential relationship between higher astigmatism and the enhancement of stereopsis after PRK. Furthermore, our study found no significant impact of ORA on the outcomes of stereopsis following PRK.

## 1. Background

Stereopsis is the ability to perceive depth based on binocular disparity between the images seen by each eye [1, 2]. It is testable in the clinic and has often been employed as the stand‐alone measure of depth perception [3–5].

In recent years, with the gradual maturation of myopia correction refractive surgery, the changes in binocular visual function after surgery have attracted widespread attention [6]. A common method of ablative refractive surgery worldwide is photorefractive keratectomy (PRK) [7–11]. Even though many patients have achieved emmetropia after refractive surgery, evaluation of the higher visual function—such as binocular vision and stereopsis—might reveal a more comprehensive assessment. While some studies report improved stereopsis after corneal refractive surgery in young adults and pediatric patients, others note a decline following the procedure [12–16].

Astigmatic correction as an essential component of refractive error could modify meridional aniseikonia and accordingly the binocular vision [17, 18]. Meridional aniseikonia causes directional image distortions that can disrupt binocular fusion and stereopsis, especially with vertical or oblique magnification [19]. Research indicates that the visual system can manage small levels of aniseikonia without completely impairing binocular fusion. However, as the severity of aniseikonia increases, stereopsis begins to deteriorate. Multiple studies have explored this relationship by examining the impact of different levels of induced aniseikonia on stereopsis, but their findings have shown significant variability [19, 20]. Ocular residual astigmatism (ORA) as a component of astigmatism may influence the outcomes of refractive surgery and postoperative stereopsis, along with other factors such as age, ocular physiology, anisometropia, nonstrabismic binocular anomalies, and higher order aberrations [21–24].

Few studies have focused on stereoacuity in relation to refractive errors, particularly astigmatism [25, 26]. The components of astigmatism, which include corneal and internal factors, may have varying effects and levels of significance. Therefore, we aimed to determine the role and relative importance of these components in enhancing stereopsis after astigmatism correction. In this study, we aimed to evaluate the impact of refractive and internal astigmatic components on stereopsis after PRK for corrections of myopia and myopic astigmatism.

## 2. Materials and Methods

### 2.1. Population and Study Design

This prospective observational study included patients who underwent PRK for the treatment of myopia and myopic astigmatism. The research was conducted at Parsian Clinic in Isfahan, Iran, adhering to the ethical principles outlined in the Declaration of Helsinki. Informed consent was obtained from all participants, and the study received approval from the Ethics Committee of Isfahan University of Medical Sciences (http://ethics.research.ac.ir/IR.MUI.MED.REC.1400.204).

The surgeries were performed bilaterally in a single session. Participants were eligible if they met the following criteria: age 18 years or older, documented stable refraction (defined as a refractive change of less than 0.5 diopters (D) for at least 1 year prior to PRK), central corneal thickness ≥ 470 µm, spherical equivalent (SE) refraction between −0.25 and −8.0 D, refractive astigmatism of ≤ 5 D, and corrected distance visual acuity (CDVA) of 20/25 or better [27].

Patients were excluded if they have any prior ocular or refractive surgeries, cataract, or signs of ectatic corneal disorders. Additionally, individuals with a history of collagen vascular disease, diabetes, or those taking medications with cholinergic or anticholinergic effects that could impact binocular or accommodative function were excluded. Monocular or amblyopic patients, as well as subjects with preoperative stereopsis worse than 480 s/arc, abnormal pupil size (outside the range of 3–7 mm in mesopic conditions), or atypical interpupillary distance (PD) (outside the range of 54–74 mm), were also excluded [5]. Cases with anisometropia ≥ 1 D in SE, binocular vision anomalies (e.g., strabismus or decompensated heterophoria), were excluded. Additionally, patients with PRK‐related complications (e.g., corneal haze, infection, severe dry eye) or those who missed follow‐up appointments were excluded from the study [5].

### 2.2. Preoperative Assessment

The preoperative ophthalmic and optometric examination included a variety of assessments. VA was measured using a Snellen acuity chart (Medizs LED chart, Smart LC 13, Korea). Refraction was evaluated through both manifest and cycloplegic methods. Anterior segment examination was conducted using slit‐lamp biomicroscopy (Topcon, SL‐D301, Japan).

Stereoacuity was assessed using the Stereo Fly Test (Stereo Optical, USA). The Stereo Fly Test is widely used in clinical practice due to its broad accessibility, ease of administration, and high patient comprehension. Unlike tests such as the Randot Stereotest or the Frisby test, the large image of the fly in the Stereo Fly Test provides an engaging and visually appealing method for patients to participate in stereopsis assessment. Studies report that the standard Stereo Fly Test protocol has a sensitivity of 81% [28, 29]. The test consists of a booklet with polarized test stimuli, designed for use at a 40 cm testing distance. The test was performed in a silent room without sunlight exposure, with standardized daylight (600 lux) and a temperature of 22°C. Participants wore test‐specific polarized glasses during the evaluation [30]. In patients with accommodative dysfunction, stereoacuity testing was performed after appropriate near‐vision correction, considering the impact of accommodative dysfunction on depth perception [31].

Corneal evaluation was conducted using Scheimpflug corneal tomography (Pentacam HR, Oculus, Germany), which provided measurements of pupil size, average keratometry, simulated keratometry (simK), total corneal astigmatism, central corneal thickness, and anterior and posterior *Q* values.

### 2.3. Surgical Technique

A single surgeon (A.P.) performed all procedures. Topical tetracaine 0.5% drops were applied for anesthesia, followed by insertion of an eyelid speculum. The surface corneal epithelium in 8.5‐mm‐diameter area was loosened using a 20% alcohol solution and removed using a blunt spatula (Hockey knife). Surgery was performed using a Schwind Amaris 1050RS excimer laser system (Schwind; Kleinostheim, Germany) with an aberration‐free ablation profile. Final refraction was determined for each case using the surgeon’s personalized nomogram (for astigmatism, the goal of treatment was total correction, and for spherical error, an overcorrection between 0 and 10% applied with regard to the age of the subjects). At the end of the procedure, an Acuvue Advance HydraClear soft contact lens (Johnson & Johnson Vision Care, Inc.) was applied to each cornea [32, 33].

### 2.4. Postoperative Protocol

After surgery, all patients received a soft bandage contact lens along with a standard postoperative regimen of antibiotics and corticosteroids. Follow‐up visits were scheduled for Day 1 and Day 7. At 6 months, patients underwent an optometric evaluation, which included assessments of stereoacuity, monocular amplitude of accommodation, and near heterophoria. No significant complications, such as corneal haze or infections, occurred that could interfere with these measurements.

### 2.5. Outcome Measures

For statistical analysis, stereoacuity values were converted into the logarithm of the second of arc to ensure greater accuracy. Based on changes in stereopsis, participants were categorized into three groups: deterioration, defined as any increase (worsening) in log‐stereoacuity; improvement, defined as any decrease (enhancement) in log‐stereoacuity; and maintenance, defined as no change in log‐stereoacuity values (i.e., changes in stereoacuity = 0).

Total refractive astigmatism was calculated as the vector summation of corneal keratometric astigmatism and ORA. ORA was defined as the vectorial difference between corneal plane refractive astigmatism and anterior corneal astigmatism:

(1) ORA=refractive astigmatism−anterior corneal astigmatism.

VA was transformed into the logarithm of the minimum angle of resolution (LogMAR). Astigmatism was categorized as “with the rule” (WTR) when the steepest corneal meridian was between 60° and 120°, and as “against the rule” (ATR) when it was between 0°–30° and 150°–180°.

### 2.6. Statistical Analysis

Sample size calculations were performed based on available data to ensure a statistical power of 80% for detecting meaningful differences in stereopsis outcomes. To eliminate interocular correlation, only the right eye of each patient was included in the analysis. Quantitative data were presented as mean ± SD and median [min–max], while qualitative data were reported as percentages. The normality of the data was assessed using the Kolmogorov–Smirnov test. Statistical comparisons of changes in stereopsis and astigmatism axis among patients were performed using the Kruskal–Wallis and chi‐square tests, as the data were not normally distributed. Data were analyzed using SPSS software (Version 25, Chicago, IL, USA). A *p* value of less than 0.05 was considered statistically significant.

## 3. Results

The study involved a total of 96 eyes from 96 patients. The patients had a mean age of 29.83 ± 7.39 years, with a median age of 29.50 years (range: 19–54 years). The majority of the participants were female, comprising 70 subjects (72.9%). The mean sphere and cylinder refractive errors were −2.97 ± 1.75 and −1.15 ± 1.09 diopters (D), respectively. Seventy patients (72.9%) had WTR, 10 patients (10.4%) had oblique, and 16 patients (16.7%) had ATR astigmatism. The mean preoperative ORA was 0.79 ± 0.35 D. Baseline demographics and clinical characteristics of the participants are presented in Table 1.

**Table 1:** Characteristics data of 96 patients (96 eyes) enrolled in study.

Variables	
Age (year)	
Mean ± SD	29.83 ± 7.39
Median [min, max]	29.50 [19, 54]
Gender f (%)	
Female	70 (72.9)
Male	26 (27.1)
Sphere (D)	
Mean ± SD	−2.97 ± 1.75
Median [min, max]	−2.75 [−7.50, 0.25]
Cylinder (D)	
Mean ± SD	−1.15 ± 1.09
Median [min, max]	−0.75 [−4.75, 0.00]
Astigmatism type f (%)	
WTR	70 (72.9)
Oblique	10 (10.4)
ATR	16 (16.7)
Interpupillary distance (mm)	
Mean ± SD	62.29 ± 3.51
Median [min, max]	61.50 [54, 73]
$\sqrt{\;X{\text{Chord}}^{2}+{Y\text{Chord}}^{2}}$	
Mean ± SD	0.19 ± 0.11
Median [min, max]	0.16 [0.01, 0.47]
Steep K	
Mean ± SD	44.60 ± 1.91
Median [min, max]	44.69 [40, 48.9]
Flat K	
Mean ± SD	43.18 ± 1.78
Median [min, max]	43.10 [39.50, 48.50]
Corneal asphericity (*Q* value)	
Mean ± SD	−0.25 ± 0.09
Median [min, max]	−0.25 [−0.49, −0.04]
CCT	
Mean ± SD	539.05 ± 30.77
Median [min, max]	539.0 [480, 630]
ORA	
Mean ± SD	0.79 ± 0.35
Median [min, max]	0.74 [0.15, 1.89]
Axis of ORA f (%)	
WTR	7 (7.3)
Oblique	7 (7.3)
ATR	82 (85.4)

*Note:* K, keratometry.

Abbreviations: ATR, against the rule; CCT, central corneal thickness; ORA, ocular residual astigmatism; SD, standard deviation; WTR, with the rule.

The mean stereoacuity before and after PRK was 1.65 ± 0.09 in log units (median [min–max]: 40 arcsec [40–140]) and 1.63 ± 0.07 in log units (median [min–max]: 40 arcsec [40–140]), respectively. However, these improvements did not reach a statistically significant level (*p* = 0.17). Based on changes in stereopsis value, 19 patients experienced deterioration, 16 showed improvement, and 61 maintained the same level.

Table 2 presents the baseline clinical and topographic variables based on the outcome of stereopsis. Patients with improvement in stereopsis had higher mean cylindrical error in comparison with other groups (*p* = 0.048) (Table 2). The average pretreatment ORA was 0.84 ± 0.37 in patients who experienced deterioration in stereopsis, 0.77 ± 0.35 in patients who maintained the same level, and 0.80 ± 0.35 in patients who showed improvement. However, this difference was not statistically significant (*p* = 0.77).

**Table 2:** Baseline clinical and topographic variables based on the outcome of stereopsis.

Variable	Stereopsis change			
	Deterioration (*n* = 19)	Maintained (no change) (*n* = 61)	Improvement (*n* = 16)	*p* value
Age (year)				
Mean ± SD	30 ± 9.26	29.58 ± 7	30.62 ± 6.80	0.790^¶^
Median [min, max]	28 [19, 47]	45 [19, 54]	30.50 [21, 42]	
Gender f (%)				
Female	17 (24.3)	43 (61.4)	10 (14.3)	0.148^©^
Male	2 (7.7)	18 (69.2)	6 (23.1)	
Pretreatment sphere (D)				
Mean ± SD	−3.80 ± 1.79	−2.89 ± 1.76	−2.26 ± 1.29	0.068^¶^
Median [min, max]	−3.25 [−7.50, −1.25]	−2.75 [−7.25, 0.25]	−2.37 [−4.00, 0.25]	
Pretreatment cylinder (D)				
Mean ± SD	−1.25 ± 1.13	−0.96 ± 1.00	−1.76 ± 1.17	0.048^¶^^∗^
Median [min, max]	−0.5 [0.1, 0.4]	−0.5 [−4.75, 0.00]	−1.62 [−3.50, 0.00]	
Pretreatment astigmatism type f (%)				
WTR	12 (17.1)	47 (67.1)	11 (15.7)	0.498^©^
Oblique	2 (20)	7 (70)	1 (10)	
ATR	5 (31.3)	7 (43.8)	4 (25)	
Pretreatment UCVA (LogMAR)				
Mean ± SD	0.16 ± 0.10	0.17 ± 0.14	0.17 ± 0.22	0.962^¶^
Median [min, max]	0.1 [0.1, 0.4]	0.1 [0.1, 0.8]	0.1 [0.1, 0.8]	
Interpupillary distance (mm)				
Mean ± SD	62.26 (4.48)	62.13 (3.16)	62.93 (3.66)	0.851^¶^
Median [min, max]	61 [54, 73]	62 [56, 68]	61 [59, 69]	
Pretreatment $\sqrt{\;X{\text{Chord}}^{2}+{Y\text{Chord}}^{2}}$				
Mean ± SD	0.17 ± 0.10	0.19 ± 0.10	0.18 ± 0.10	0.781^¶^
Median [min, max]	0.15 [0.03, 0.37]	0.16 [0.01, 0.47]	0.16 [0.01, 0.41]	
Pretreatment steep K				
Mean ± SD	44.31 ± 1.43	44.63 ± 2.00	44.81 ± 2.12	0.702^¶^
Median [min, max]	44.26 [42.20, 47.20]	44.45 [40.00, 48.90]	45.20 [41.40, 48.90]	
Pretreatment flat K				
Mean ± SD	42.95 ± 1.36	43.32 ± 1.90	42.91 ± 1.79	0.527^¶^
Median [min, max]	42.70 [41.16, 46.00]	43.34 [39.50, 48.50]	42.70 [39.70, 46.10]	
Pretreatment corneal asphericity				
Mean ± SD	−0.23 ± 0.11	−0.25 ± 0.96	−0.26 ± 0.09	0.739^¶^
Median [min, max]	−0.23 [−0.47, −0.04]	−0.25 [−0.49, −0.04]	−0.26 [−0.41, −0.13]	
Pretreatment CCT				
Mean ± SD	545.26 ± 31.81	538 ± 26.67	534.87 ± 34.40	0.681^¶^
Median [min, max]	545 [502, 630]	540 [481, 615]	525.50 [479, 601]	
Pretreatment ORA				
Mean ± SD	0.84 ± 0.37	0.77 ± 0.35	0.80 ± 0.35	0.766^¶^
Median [min, max]	0.79 [0.34, 1.89]	0.73 [0.15, 1.82]	0.75 [0.31, 1.52]	
Pretreatment axis of ORA f (%)				
WTR	2 (28.6)	5 (71.4)	0 (0.0)	^©^0.189
Oblique	0 (0.0)	4 (57.1)	3 (42.9)	
ATR	17 (20.7)	52 (63.4)	13 (15.9)	

*Note:* K, keratometry.

Abbreviations: ATR, against the rule; CCT, central corneal thickness; ORA, ocular residual astigmatism; SD, standard deviation; WTR, with the rule.

^¶^Result from the Kruskal–Wallis test.

^©^Result from chi‐square test.

^∗^Indicates statistical significance (*p* < 0.05).

Table 3 presents the baseline clinical and topographic variables based on the axis of astigmatism, comparing those of WTR (*n* = 70) to those of others (ATR/oblique) (*n* = 26). Significant differences were found in age. Patients with WTR astigmatism were younger, with a mean age of 29.0 ± 7.67 compared to 32.09 ± 6.18 years in the ATR/oblique group (*p* = 0.03). Additionally, statistically nonsignificant was reported in two factors: steep K (*p* = 0.06) and preoperative ORA (*p* = 0.099) (Table 3).

**Table 3:** Baseline clinical and topographic variables based on astigmatism type.

Variable	Astigmatism type		
	WTR (*n* = 70)	ATR/oblique (*n* = 26)	*p* value
Age (year)			
Mean ± SD	29.0 ± 7.67	32.09 ± 6.18	0.033^¶^
Median [min, max]	27.0 [19, 54]	32 [19, 45]	
Gender, f (%)			
Female	54 (77.1)	16 (22.9)	0.126^©^
Male	16 (61.5)	10 (38.5)	
Pretreatment sphere (D)			
Mean ± SD	−3.07 ± 1.88	−2.69 ± 1.76	0.426
Median [min, max]	−3.0 [−7.50, 0.25]	−2.5 [−5.75, 0.0]	
Pretreatment cylinder (D)			
Mean ± SD	−1.27 ± 1.18	−0.85 ± 0.73	0.223^¶^
Median [min, max]	−1.0 [−4.75, 0.0]	−0.50 [−3.0, −0.25]	
Pretreatment UCVA (LogMAR)			
Mean ± SD	0.73 ± 0.40	0.63 ± 0.33	0.415^¶^
Median [min, max]	0.70 [0.1, 2.3]	0.52 [0.22, 1.30]	
Interpapillary distance (mm)			
Mean ± SD	61.97 ± 3.21	63.015 ± 4.17	0.177^¶^
Median [min, max]	61.0 [56.0, 69.0]	65.5 [54, 73]	
$\text{Pretreatment}\sqrt{\;X{\text{Chord}}^{2}+{Y\text{Chord}}^{2}}$			
Mean ± SD	0.19 ± 0.11	0.17 ± 0.08	0.499^¶^
Median [min, max]	0.17 [0.01, 0.47]	0.159 [0.04, 0.37]	
Pretreatment steep K			
Mean ± SD	44.8 ± 1.96	44.05 ± 1.69	0.064^¶^
Median [min, max]	44.95 [40, 48.9]	43.74 [40.2, 48.2]	
Pretreatment flat K			
Mean ± SD	43.15 ± 1.84	43.27 ± 1.66	0.779^¶^
Median [min, max]	43 [39.5, 48.5]	43.15 [39.54, 47.38]	
Pretreatment *Q* value			
Mean ± SD	−0.25 ± 0.10	−0.26 ± 0.08	0.789^¶^
Median [min, max]	−0.25[−0.49, −0.04]	−0.26 [−0.41, −0.08]	
Pretreatment CCT			
Mean ± SD	538.8 ± 28.82	539.6 ± 36.32	0.970^¶^
Median [min, max]	538.5 [488, 615]	539 [479, 630]	
Pretreatment ORA			
Mean ± SD	0.76 ± 0.37	0.86 ± 0.31	0.099^¶^
Median [min, max]	0.73 [0.15, 1.89]	0.86 [0.36, 1.52]	
Pretreatment axis of ORA			
With the rule	57 (69.5)	25 (30.5)	0.108^©^
Against the rule	6 (85.7)	1 (14.3)	
Oblique	7 (100.0)	0 (0,0)	

*Note:* K: keratometry.

Abbreviations: ATR, against the rule; CCT, central corneal thickness; ORA, ocular residual astigmatism; SD, standard deviation; WTR, with the rule.

^¶^Result from the Kruskal–Wallis test.

^©^Result from chi‐square test.

## 4. Discussion

The findings of the present study indicate that, overall, PRK does not lead to a significant change in stereopsis. However, it was observed that patients with higher degrees of astigmatism tend to experience an improvement in stereopsis. Furthermore, the average pretreatment ORA did not significantly correlate with stereopsis. These results suggest that while PRK may not substantially impact stereopsis for most individuals, it can yield positive effects on stereopsis in patients with higher astigmatism.

This study found that approximately 64% of patients experienced no change in stereopsis after undergoing PRK. Previous studies have presented conflicting findings regarding the impact of corneal refractive surgery on stereopsis. A recent investigation by Peyman et al. demonstrated a significant improvement in stereopsis following different types of corneal refractive surgeries [16]. Conversely, another study indicated that despite an overall enhancement in mean stereopsis after LASIK, a notable portion of patients exhibited a decrease in stereoacuity [13]. Zarei‐Ghanavati et al. also reported that PRK effectively and safely improved refractive error and VA; however, it had the potential to deteriorate stereoscopic vision, potentially attributed to an increase in higher order aberrations [14]. It is important that the current study employed PRK rather than LASIK, which may explain the discrepancies between the current results and previous studies. Additionally, we applied a precise surgical protocol, enforced strict patient inclusion criteria, and provided thorough follow‐up. These measures minimized the possibility of corneal opacification or epithelial irregularities contributing to stereoacuity deterioration.

Astigmatism is a key factor that significantly influences refractive error and VA. In our study, patients who experienced improvement in stereopsis after undergoing PRK had a higher mean level of astigmatism. This finding aligns with a study by Singh et al., which demonstrated that a greater amount of astigmatism correction resulted in improved stereoacuity following surgery [34]. Astigmatic correction is crucial in refractive error correction, as it can modify meridional aniseikonia and significantly impact binocular vision [9, 35]. By reducing image size disparities between the eyes, astigmatic correction may improve binocular fusion and depth perception. This effect is particularly relevant in cases of high astigmatism, where uncorrected meridional aniseikonia can substantially disrupt binocular vision.

Our study findings indicate that ORA did not have any significant effect on the outcomes of stereopsis after PRK. PRK effectively corrected total refractive astigmatism by addressing both keratometric astigmatism and making additional vectorial adjustments to compensate for nonkeratometric ocular astigmatism. This suggests that PRK alters keratometric astigmatism to offset the effects of nonkeratometric astigmatism, leading to changes in front corneal astigmatism with a reduced impact of nonkeratometric astigmatism [27, 36].

Our study findings indicate the significant association between pretreatment cylinder and stereopsis improvement suggests that patients with higher astigmatism may experience greater enhancements in binocular function following PRK. This could be attributed to the fact that uncorrected astigmatism often leads to blurred vision, which may negatively impact depth perception. However, further research involving patients with varying degrees of astigmatism (mild to severe) and types of astigmatism is necessary to validate these findings.

Several factors may explain the lack of correlation between ORA and postoperative stereoacuity. First, it is possible that the changes in stereopsis after surgery were limited, thus minimizing the potential influence of ORA. Additionally, the effect of ORA may have been too small to detect given our sample size. When a parameter has a minor effect or cannot be accurately measured, it may not be clinically significant. Recent evidences suggest that the visual system adapts to astigmatic blur in the short term, which may reduce the perceived impact of astigmatism on vision [1, 2]. This adaptation may contribute to the absence of a connection between ORA and stereopsis in our findings. Furthermore, the long follow‐up period might have affected the relationship between ORA and stereopsis, potentially warranting further investigation. Overall, the lack of association between pretreatment ORA and stereopsis outcome in our study could be attributed to the limited changes in stereopsis postsurgery, the potential small effect of ORA, and short‐term adaptations to astigmatic blur.

The current study has notable strengths, including the comprehensive evaluation of various parameters, including ORA, that have the potential to influence stereoacuity. Additionally, the study benefited from a relatively long‐term follow‐up period and a sufficient sample size compared to previous studies.

However, there are several limitations to consider. First, the study relied on simulated keratometry readings from the tomography for calculations, which may not accurately represent the true corneal effect on refraction when considering the modified calculation of power based on front curvature. This could introduce a source of error in the findings. It should be noted that the stereoacuity test has only 81% sensitivity, and a deterioration or no change in stereoacuity should be interpreted with caution due to the possibility of an undetected actual improvement. Furthermore, the lack of a significant difference in stereopsis before and after surgery may be attributed to the small sample size within the astigmatism types (ATR and oblique subgroups). The population included in this study predominantly consisted of individuals with a myopic SE. As a result, it is plausible that the absence of an effect of ORA on stereopsis in this study can be attributed to the limited interference of myopic astigmatism with stereoacuity. It is known that binocular myopic astigmatism has the potential to enhance near vision [37]. Additionally, evaluating contrast sensitivity and wavefront aberrometry incorporating higher order aberration analysis would provide valuable data for further investigations in this field.

## 5. Conclusion

This study found that stereopsis did not significantly change after PRK. However, patients with higher mean astigmatism experienced statistically significant improvements in stereopsis, suggesting that greater astigmatic correction may offer additional functional benefits beyond enhanced VA. No association was observed between pretreatment ORA and changes in stereopsis. While certain topographic variables showed marginal associations with astigmatism type, their clinical significance remains uncertain and warrants further investigation. PRK remains an effective refractive correction, and its potential impact on stereopsis should be further explored in larger studies and more detailed assessments of binocular visual function.

**Nomenclature**
PRK: Photorefractive keratectomy
ORA: Ocular residual astigmatism
CDVA: Corrected distance visual acuity
VA: Visual acuity
LogMAR: Logarithm of the minimum angle of resolution
WTR: With the rule
ATR: Against the rule

## Ethics Statement

The protocol of the study was approved by the Institutional Ethics Committee of IUMS, Isfahan, Iran (http://ethics.research.ac.ir/IR.MUI.MED.REC.1400.204). After an adequate explanation of the project, informed consent was obtained before the entry of the study patients.

## Consent

Please see the Ethics Statement.

## Disclosure

All authors revised it critically and have approved the final version. The authors take full responsibility for the content.

## Conflicts of Interest

The authors declare no conflicts of interest.

## Author Contributions

Alireza Peyman and Faezeh Fayaz contributed to the study design. Alireza Peyman, Faezeh Fayaz, Payam Nabovati, and Mohsen Pourazizi performed the clinical study. Alireza Peyman, Mohsen Pourazizi, and Pegah Noorshargh analyzed and interpreted the data. Faezeh Fayaz wrote the first draft. Payam Nabovati and Pegah Noorshargh amended the manuscript.

## Funding

The authors have nothing to report.

## Data Availability

The data used to support the findings of this study are available from the corresponding author upon request.
